# Bread Improvement with Nutraceutical Ingredients Obtained from Food By-Products: Effect on Quality and Technological Aspects

**DOI:** 10.3390/foods13060825

**Published:** 2024-03-07

**Authors:** Giulio Scappaticci, Nicola Mercanti, Ylenia Pieracci, Corrado Ferrari, Roberto Mangia, Andrea Marianelli, Monica Macaluso, Angela Zinnai

**Affiliations:** 1 Department of Agriculture, Food and Environment, University of Pisa, Via del Borghetto 80, 56124 Pisa, Italy; giulio.scappaticci@phd.unipi.it (G.S.); nicola.mercanti@phd.unipi.it (N.M.); r.mangia@studenti.unipi.it (R.M.); monica.macaluso@unipi.it (M.M.); angela.zinnai@unipi.it (A.Z.); 2 Department of Pharmacy, University of Pisa, Via Bonanno 6, 56124 Pisa, Italy; 3 R&D Bakery Department, Barilla G e R Fratelli, Via Mantova 166, 43122 Parma, Italy; corrado.ferrari@barilla.com; 4 Interdepartmental Research Centre “Nutraceuticals and Food for Health”, University of Pisa, Via del Borghetto 80, 56124 Pisa, Italy

**Keywords:** dough improvement, bakery products, shelf-life, pectin, grape pomace

## Abstract

The use of by-products as functional ingredients for bread recipes may open up new horizons in terms of product innovation to increase nutraceutical characteristics and/or shelf-life. In this research, the ability of residual products from important food chains (*Citrus* and wine) to influence the water binding capacity of dough and bread was investigated in order to create industrial breads of high quality with prolonged shelf-life in the absence of any chemical additives (e.g., ethanol, sorbic acid, and propionic acid). The product under study is the ‘Pan Bauletto bianco con olio EVO’ (white bakery bread with EVO oil), an ‘industrial bread’ type usually treated with ethanol before being marketed, aiming to prolong its short shelf-life. The effect of the addition of different amounts of pectin (*Citrus* supply chain) and grape pomace (wine supply chain), in combination or not, has shown promising results from both a technological point of view and the increasing shelf-life, allowing to obtain products with high nutraceutical value and interesting properties.

## 1. Introduction

Cereals are the raw materials for the production of bakery products. They represent the edible seeds of plant species belonging to the Gramineae family, which is one of the most cultivated in the world. Generally, both soft (*Triticum aestivum*) and durum (*Triticum durum*) wheat flours are used for the production of bread and baked goods. The realm of baking underwent a significant transformation with the introduction of commercially available yeast strains. Historically, bread fermentation relied on either sourdough, lending tangy notes, or the frothy residue (barm) from beer brewing, offering a bitter, hoppy essence. Presently, the most prevalent and straightforward form of natural leavening involves brewer’s yeast—compressed blocks containing specific strains of *Saccharomyces cerevisiae*. Nevertheless, there’s a growing interest in food fermentation using sourdough, driven by its perceived nutritional advantages [1], since consumers are increasingly demanding food products with high nutritional value [2,3,4].

Food fortification stands out as one of the most sustainable and cost-effective approaches to improving public health [5,6,7]. Establishing tolerable upper intake levels, also known as safe upper intake levels, is crucial when devising a food fortification strategy, allowing for the assessment of the risk of excessive intake of individual micronutrients [8].

Current research actively explores natural agents capable of enhancing the baking properties of flour and processing solutions. One noteworthy compound with high water absorption is pectin. Pectin constitutes structural carbohydrates found within plant cell walls, synthesized in Golgi vesicles. Initially fully esterified during synthesis, pectin undergoes ester bond cleavage by the enzyme pectin methyl esterase upon reaching target cells. Within plant cells, pectin serves to form the middle lamella, imparting adhesion and elasticity.

While traditional sources for pectin extraction include apple and citrus peels, by-products of juice production, recent investigations have explored new extraction sources such as sugar beet, potato, sunflower, and papaya. These different materials contain substantial quantities of pectic substances, each possessing distinct chemical characteristics suitable for specific applications [9]. In the context of breadmaking, the current literature presents divergent findings regarding the incorporation of pectin during dough preparation, optimal dosages within formulations, and methods of dough preparation. Various experiments suggest that pectin, along with hydrocolloids in general, enhances bread volume, imparts a softer texture, and mitigates chilling effects. Additionally, they reduce physical damage caused by ice crystal formation in bread produced from partially baked frozen dough [10]. Polyphenols present in bread formulations originate from raw materials and intermediates formed during baking processes (e.g., Maillard reaction products), as well as from heat-induced degradation products of polyphenols and polyphenol-polysaccharide/protein complexes. The complexation between polyphenols and proteins/polysaccharides occurs through hydrogen bonding and/or hydrophobic interactions, influenced by factors such as molecular size, mobility, solubility, and structural characteristics of polyphenols, proteins, and polysaccharides. Furthermore, the presence or absence of other bread ingredients further modulates these interactions [11]. Functional diets, including those rich in dietary fiber and polyphenols, have been associated with reduced risks of various diet-related ailments, such as diabetes, cardiovascular disease, obesity, hypertension, and gastrointestinal disorders. Cereal products, notably bread, serve as staple foods that offer avenues for imparting health benefits to broad populations. Consequently, bread emerges as a primary candidate for fortification with functional components [12]. In the realm of dough food ingredients, pectin and red grape skins exhibit favorable characteristics such as water-holding capacity, high water binding ability, and effective gelling properties [13].

Utilizing by-products from the food industry as functional ingredients for incorporation into traditional bread recipes holds promise for product innovation aimed at enhancing nutraceutical attributes and/or extending shelf life. In the present study, conducted in collaboration with Barilla company, the potential utilization of residual materials from significant food chains (*Citrus* and wine) was explored to develop high-quality industrial breads with prolonged shelf life without the addition of chemical additives (e.g., ethanol, sorbic acid, and propionic acid). The objective of this investigation, conducted at the Food Technology laboratory within the Department of Agriculture, Food, and Environment (DAFE) in Pisa, was to evaluate the impact on dough water binding capacity and resultant bread staling prevention through the incorporation of different levels of pectin into the dough. Changes in relative and absolute humidity throughout the entire bread-making process, from proofing to bread cooling, were monitored to assess the effect of pectin addition.

This fortification approach relied on the water-binding capability of hydrocolloids, including pectin, to modify dough characteristics and, consequently, the quality of the final product. Additionally, red grape pomaces, discarded from the wine vinification processes of a prominent winery in the Maremma region (La Cura), were utilized due to their high fiber and phenolic compound content. These pomaces were employed either alone or in conjunction with pectin. The focus was on the ‘Pan Bauletto bianco con olio EVO’ (white bakery bread with EVO oil), categorized as an ‘industrial bread’ characterized by a thin crust and a crumb featuring regular porosity and thin-walled, parallel-piped shaped cells. The inclusion of fat in the formulation imparts a soft and elastic consistency to the bread. However, this product typically exhibits a short shelf life due to factors such as uncontrolled storage conditions (ambient temperature and relative humidity). To address this challenge, in industrial settings, such products are often surface treated with ethanol prior to being introduced to the market.

## 2. Materials and Methods

### 2.1. Materials

The bread was produced with type 0 wheat flour, malted barley flour, water, apple vinegar, extra virgin olive oil, compressed brewer’s yeast, enzymes, and salt. During the preparation of the bread, *Citrus* pectin (E440 FARMALABOR Assago, Milan, Italy) in 2% concentration *w*/*w*, 2% *w*/*w* of lyophilized red wine pomace powder, and a combination of 2% *w*/*w* of *Citrus* pectin and 2% *w*/*w* of lyophilized red wine pomace powder were added to the dough. All of these ingredients were added, replacing the correspondent aliquots of wheat flour. The lyophilization of the grape pomaces was performed using the lyophilizer LyoQuest (Telsar, Terrassa, Spain).

### 2.2. Breadmaking

The control samples were prepared using a kneading machine (model SV5, 1 speed, 1400 rpm, Officine Meccaniche Sangrigoli, Giarre, Italy). Wheat flour, malted barley flour, enzymes, water, apple vinegar, brewer’s yeast previously dissolved in a portion of water, extra virgin olive oil, and salt were added and mixed for approximately 10 min until the dough reached the desired consistency. The dough was then allowed to rest for 10 min at room temperature, divided into portions weighing 460 g each, and left to rest for an additional 10 min at room temperature. Subsequently, all portions of dough were shaped, placed in steel molds, and left to leaven in a proving chamber (model FOALSTR23M, Fimar S.p.A., Villa Verrucchio, Italy) at 38 °C and 86% relative humidity for 70 min. Following this, the bread samples were baked in an oven (model FOSTR1040T, Fimar S.p.A., Villa Verrucchio, Italy) at 210 °C for 21 min. Once baked, the breads were cooled under a laminar flow hood (model Olympia 1.2, Bioair instruments S.r.l., Siziano, Italy) for 2 h.

The fortified breads were produced following the same procedure as the control bread, with some modifications. Pectin was emulsified with extra virgin olive oil, which was then dissolved in an aliquot of water and added to the dough during the mixing phase. Grape pomace powder, on the other hand, was directly incorporated into the dough. After the breadmaking process, the samples were packed into polyethylene bags filled with either technical air or a modified atmosphere (MAP) composed of 70% *v*/*v* CO_2_ and 30% *v/v* Argon. The samples were stored at room temperature (20–24 °C) and 40% relative humidity.

### 2.3. Measurement of Dough Development

An aliquot of 20 g of dough from each of the different recipes was taken, inserted into a graduated cylinder, and left to rise in the proving chamber for the proofing time described in Section 2.2. The volume of the dough was measured at the beginning of the rising process and when the process was over. The variation in volume was calculated using the following formula:

$$Variation\left(\%\right)=\frac{V_{f}-V_{0}}{V_{0}}\;*\;100$$

where V_0_ is the volume of 20 g of dough at the beginning of the leaving phase and V_f_ is the volume of the dough at the end of the leaving time. The dough development has been calculated with the equation previously reported by Bianchi et al. [14].

### 2.4. Physical-Chemical Characterization of Samples

The samples were weighed daily using a 10 mg resolution analytical balance until the conclusion of their shelf-life. The termination point of the samples’ shelf-life was determined by the emergence of the first visible microbial colony. Physical-chemical parameters, including moisture content, pH, and free acidity, were assessed utilizing the methods outlined by Bianchi et al. [14].

### 2.5. Activity Water Measurement

Before the analysis, the samples were cut with a bread slicer (model smart 42, Mecnosud S.r.l., Valle Ufita, Italy) in slices of 1 cm thickness. The activity water was measured on the crumb prevailed from the center of the slices by means of an a_w_ meter (model HygroPalm HP23-AW-A, Rotronic AG, Bassersdorf, Switzerland).

### 2.6. Penetrometric Index

The compressibility of the bread slices was evaluated using a PNR-12 penetrometer (Anton Paar, Rivoli, Italy). In detail, two slices from the center of the bread were measured into three different spots with a weight of 40 g for 5 s. The results are expressed in millimeters of penetration (0.1 mm corresponds to 1 penetration unit).

### 2.7. Color Determination

Crumb color was measured on two central slices with a colorimeter (model CLM-196 Benchtop, Eoptis, Trento, Italy) expressing the measurement following the CIE L*a*b* color system, where the color is defined in relation to the chromatic coordinates brightness (L*), red-green range (a*), and blue-yellow range (b*). The Chroma (C*) and tint (H*) values are expressed by the following equations:

$$C^{*}=2a^{*}+2b^{*}$$

$$H^{*}=\arctan\left(a^{*}\;*\;b^{*}\right)$$

The color difference between samples (${∆E}_{ab}^{*}$) is expressed by the equation:

$${∆E}_{ab}^{*}\sqrt{({∆L}^{*}{)}^{2}+(∆a^{*}{)}^{2}+(∆b^{*}{)}^{2}}$$

### 2.8. Total Phenolic Evaluations

The extractions for the evaluation of total phenolic content were performed following the methods reported [15].

After the extraction, the total phenolic content was determined with the Folin-Ciocâlteu method according to [16].

### 2.9. Ethanol and Simple Sugars (Glucose and Fructose) Contents

Ethanol and simple sugar concentrations were estimated by enzymatic kit analysis, as reported by Taglieri et al., 2021 [15]. All the enzymatic kits were supplied by Megazyme Ltd. (Wicklow, Ireland).

### 2.10. Statistical Analysis

All physicochemical analyses were conducted in triplicate, and the reported data represent the means of the individual assessments. Statistically significant differences among the samples were evaluated using one- or two-way ANOVA (CoStat, Cohort 6.0), with the homogeneity of variances assessed using the Bartlett test. A probability of *p* ≤ 0.05 indicates that the variances may not be homogeneous. The significance level of the variants is denoted by: *** if *p* < 0.001, ** if *p* < 0.01, * if *p* < 0.05, ns (not significant) if *p* > 0.05, and nd (not detected).

Data obtained from sensors and chemical parameters during sample conservation were analyzed using ROOT software 6.28, as described by Brun & Rademakers [17].

## 3. Results

Physical-chemical analyses were performed after the cooling process of the samples, specifically after two h under a microbiological hood with laminar flow.

### 3.1. Volume Increasing in Leaving and Dough Density

The results of volume-increasing doughs, expressed as means ± confidence range, are reported in Table 1.

The analysis of variance revealed strong, significant differences in the leaving volume among doughs (*p* < 0.001). Notably, the volumetric variation during the leaving time under controlled conditions of humidity (UR) and temperature resulted higher in the bread produced with the addition of pectin within the dough at concentrations of 2% than in all the other samples. Indeed, the doughs were more stable and able to support a greater extension in leavening, causing a different structure of alveolation in the final product. Pectin is able to create hydrophilic complexes with gluten proteins, and the capacity of complexation appears to be related to the density of the anionic group in the polysaccharide [18].

Conversely, no significant differences were found in the volume variation of leavening among the other samples (C, M2, and M2P2). The obtained results represented a positive outcome since the pomace of red wine added to the dough did not determine a significant variation in fermentative activity compared to the control. The density of dough (150 g) prepared following the different recipes was also measured, and density values, expressed as means ± confidence ranges, are reported in Table 2.

Additions of pectin in concentrations of 2% *w/w* to the dough resulted in a statistically significant lower bread density compared to samples prepared without pectin (M2) or to the one prepared with both pectin and red grape pomace powder (M2P2) (*p* < 0.01). The reduced values of this parameter can be explained by the higher volume of the samples after cooking as well as by the major volumetric increase during the proofing phase.

### 3.2. Moisture of the Dough and Bread Samples

The concentrations of dry matter and water were determined in the different doughs and breads. The data for these two parameters are reported in Table 3 and Table 4, respectively.

Statistical analysis evidenced a significant influence (*p* < 0.001) of the different recipes on both dry matter and water content. Indeed, the investigated parameters showed the opposite trend, as dry matter resulted highest in the doughs produced by the addition of 2% of pectin (P2) alone or in combination with grape pomace powder (M2P2), both showing the lowest water content. Conversely, the control (C) and the doughs with grape pomace powder (M2) showed the lowest dry matter and the highest water content without showing significant differences.

The dry matter and water content of the breads produced with the different recipes did not show significant differences, probably due to the cooking conditions that should be responsible for the removal of the effects caused by the different compositions of the doughs.

### 3.3. pH and Tritable Acidity

All the different additions to the control recipe determined a reduction of the pH value and an increase of the titratable acidity (TTA) (Table 5).

Analysis of variance showed that the addition of pectin to the dough causes a dose-dependent decrease in pH (*p* < 0.001). Interestingly, the pH value achieved with the addition of both pectin and grape pomace powder was the lowest; the synergistic acidification effect explicated by these two ingredients on the final product was greater compared to the two ingredients added individually as well as to the control bread.

### 3.4. Penetrometric Index

The penetrometric index defines the penetration resistance given by the bread slice. The penetration indices of the different bread samples are reported in Table 6.

Since the analysis of variance did not show any statistical differences among the samples, it was possible to affirm that the addition of both pectin and grape pomace to the control recipe did not influence neither the softness nor the elasticity of the analyzed slices of bread.

### 3.5. Activity Water

Water activity (a_w_), along with pH, is an important parameter that influences the shelf-life of bread. In Table 7, the values of a_w_ and their confidence ranges related to the samples with different recipes are shown.

As reported in Table 7, control samples exhibited statistically comparable water activity values to samples M2 and M2P2 produced with grape pomace alone or in combination with pectin. Conversely, the addition of only pectin to the dough determined significant variations in this parameter (*p* < 0.001). This aspect may represent an important aspect for further studies aiming at extending the bread’s shelf-life.

### 3.6. Phenolic Content in Dough and Bread Samples

The phenolic content was investigated in the dough at the end of the leavening time and in the bread after the cooking process in order to determine the influence of the heating treatment.

As emerged from the data reported in Table 8, the phenolic concentration was not affected by the cooking phase. Therefore, in both dough and bread, the synergic addition of grape pomace powder and pectin (M2P2) was associated with the greatest content of these metabolites, resulting in the best solution to improve the concentration of this nutraceutical fraction.

### 3.7. Ethanolic Concentration in Dough Samples before and after Proofing Time

Ethanol concentrations were evaluated by enzymatic assays before and after proofing time to determine the influence of the ingredients in the recipes on the leavening. Results are reported in Table 9, and the term “pre” indicates the ethanol concentration of the sample at the end of the molding phase, which is 20 min after the mixing process, and “post” indicates the concentration at the end of the proofing time in the white chamber with 86% UR and 38 °C.

In general, the ethanol content was highest at the end of the leavening time, and the percentage variation showed significant differences among the different recipes (*p* < 0.001), resulting in the recipe involving the addition of 2% of pectin, followed by the one with both pectin and grape pomace powder. The presence of this compound before the proofing phase, instead, was explained by the already started fermentation activity determined by the yeast inoculum made 20 min before the molding phase.

### 3.8. Simple Sugars Concentration in the Final Product

The concentration of simple sugars was evaluated in bread samples since these molecules are able to influence the sensory characteristics and the shelf-life of the final product. The concentrations of total hexose (g/kg dm) in the bread samples produced with the different formulations are reported in Table 10 and are expressed as means ± confidence ranges (n = 3).

The analysis of variance showed significant differences among the samples (*p* < 0.001). The hexose concentration of P2 was the highest, reaching concentrations almost double that detected in the control. Conversely, the breads produced with the addition of grape pomace, alone or in combination with pectin (M2 and M2P2), showed a lower total sugar content.

### 3.9. Colorimetric Analysis of Bread Samples

Breads produced following the different recipes were compared chromatically through colorimetric analysis.

**Figure 1:**
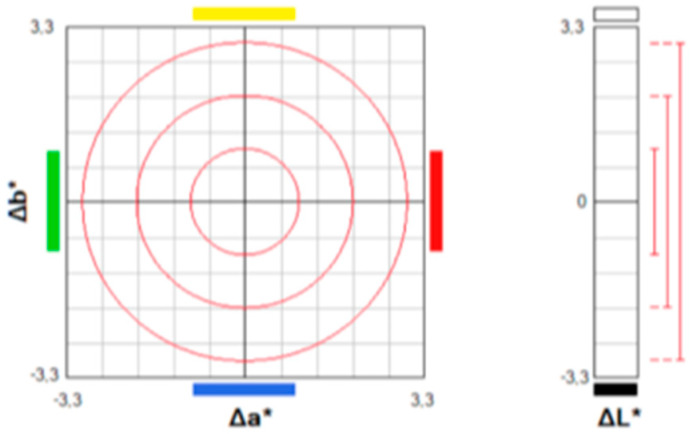
L*, a*, b* coordinates for colorimetric evaluation.

**Figure 2:**
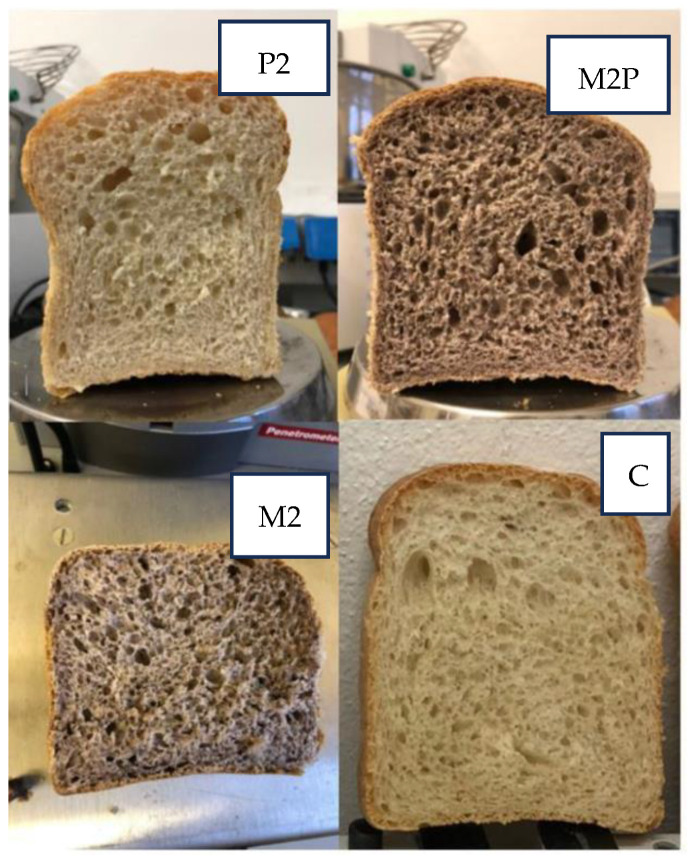
Different colors of crumb on breads made following different recipes.

Results of the analysis (Figure 1) evidenced that the addition of lyophilized red grape pomace and pectin significantly influenced the crumb color, as also visible in Figure 2.

The color coordinates of the different samples are reported in Table 11. The analysis of variance highlighted significant differences (*p* < 0.001) in the luminosity values indicated with L*, resulting greater in the pectin-added bread followed by the control one than in those produced with the addition of grape pomace powder. Conversely, chromatic coordinate a* (green-red) was also significantly influenced by the bread recipe (*p* < 0.001) and was highest in the grape pomace-enriched mixtures. Finally, regarding the blue-yellow color coordinate (b*), the C and P2 recipes have comparable higher values compared to M2 and M2P2, which in turn did not show differences between them. Color differences (ΔE*ab) among the samples are reported in Table 12.

The parameter ΔE*, which defines the existence or not of visual color variations, may express some assumptions. Considering the comparison of the different recipes to the control bread, P2 showed a barely distinguishable color variation (ΔE = 2), while M2 and M2P2 presented a completely different color, since ΔE > 12. Comparing the samples produced with the grape pomace powder, a strong colorimetric variation was observed, although in the same color range (ΔE between 6 and 12). Finally, comparing breads with pectin and grape pomace, both M2P2 and P2 and M2 and P2 showed different colors.

## 4. Discussion

The present scientific research deals with the enhancement of bakery products technological properties and shelf-life through the strategic incorporation of pectin and red grape pomace as functional ingredients. The comprehensive investigation covers various parameters, including leavening volume, bread density, pH, dry matter, water content, water activity, phenolic concentration, ethanol content, and total hexose concentration. The examination of various bread attributes highlights the complexity of the factors influencing the final product [15].

The results underscore the positive impact of pectin on both leavening volume and bread density, which represent important attributes in breadmaking. Pectin is the structural carbohydrate of the plant cell walls. Chemically, they are water-soluble heteropolysaccharides classified as dietary fiber since they are not digested and absorbed in the human small intestine [19]. However, they constitute a substrate for microbiota, determining favorable effects on human health and thus resulting in an important functional food [20]. In the food industry, pectins are widely employed as gelling agents, stabilizers, and thickeners due to their ability to form aqueous gels, modifying texture in different food systems [21]. Despite the extensive use of pectin in the food sector, these natural additives are not commonly employed in breadmaking [19]. In the present study, the improvement of leavening volume and density in the breads produced with the addition of pectin may be attributed to their ability to form hydrophilic complexes with gluten proteins [15]. Indeed, the addition of pectin has demonstrated the enhancement of the macromolecular aggregation of gluten and, thus, of the rheological properties of the bakery product by inducing a dense network of gluten [22]. The study further reveals a synergistic effect when combining pectin and red grape pomace, particularly evident in pH levels and phenolic concentration. Grape pomace represents a high-value by-product of the wine industry due to its relevant content of phenolic compounds [23]. Thus, the re-employment of those agro-industrial wastes, besides determining an improvement in some technological characteristics in breadmaking, perfectly fits with sustainable practices to pursue a circular economy. Interestingly, the reduction of pH and the improvement of phenolic content are more influenced by the association between pectin and grape pomace than the addition of the single ingredients themselves. Since pectins are natural polymers containing galacturonic acid units [24], they are probably responsible for the reduction of the pH values, which are also observed in breads produced with the higher percentages of those natural additives. In turn, the increased acidity is related to the greater phenolic content of the product obtained with both pectin and grape pomace than that produced only with grape pomace, which are the source of those bioactive compounds, since it has been demonstrated that these molecules are not stable at high pH levels [25]. Furthermore, the formulation of bread with the addition of both pectin and grape pomace leads to the best outcomes, as they allow to obtain a product with a homogeneous structure and an increased volume, with positive technological aspects if compared to the control. Moreover, considering the nutritional value, this formulation shows high phenolic content and a lower simple sugar concentration in the final product, confirming that grape pomace powder could be an attractive ingredient used to obtain fortified bread, as previously reported by Tolve et al., 2021 [26].

Notably, the research expands beyond traditional quality parameters, delving into nutritional aspects such as phenolic concentration, ethanol content, and total hexose concentration. These findings contribute to the evolving discourse on fortifying bakery products, not only for improved sensory appeal but also for potential health benefits. The exploration of color attributes provides additional insights into the visual appeal of enriched bread products. The color of food is not only determined by its colorants but also by the lighting. Many studies have shown that the light source changes the color appearance of the food and, as a consequence, influences the visual evaluation of human consumers as well as their hedonic impression [27].

In conclusion, while the study advances our understanding of the potential benefits of incorporating pectin and red grape pomace in breadmaking, it underscores the importance of continued research to fill existing knowledge gaps. The comprehensive nature of the investigation positions this study as a significant contribution to the scientific exploration of fortified bakery products.

## 5. Conclusions

The present study advances our understanding of the potential benefits of incorporating pectin and red grape pomace in breadmaking, highlighting the importance of continued research to overwhelm the existing gaps of knowledge. The study not only deals with the obtainment of a higher technological quality of bakery products but also with the improvement of the nutraceutical value of these products, which represent one of the main sources of carbohydrates. The integration *Citrus* peel and grape pomace in the bread recipe allows for the valorization of food by-products, which otherwise could become waste material and thus a cost for the industry and for the planet. The results underscore the positive impact of pectin on both leavening volume and bread density, which represent important attributes in breadmaking. In turn, grape pomace constitutes a high-value by-product of the wine industry due to its relevant content of phenolic compounds. Interestingly, the formulation of bread with the addition of both pectin and grape pomace leads to the best outcomes, as they allow to obtain a product with a homogeneous structure and an increased volume, with positive technological aspects if compared to the control.

## Figures and Tables

**Table 1:** Volume increase of 20 g of doughs with different recipes after leaving time.

Sample	Variation (%)
C	230 ± 10 ^b^
P2	268 ± 6 ^a^
M2	230 ± 5 ^b^
M2P2	241 ± 3 ^b^

Superscript lowercase letters indicate statistically significant differences among the samples. The acronyms C, P, M, and MP indicate the control dough and the doughs produced with pectin, marcs, and pectin and marcs, respectively.

**Table 2:** Density (g/cm^3^) of 150 g of dough prepared with different recipes.

Sample	Density (g/cm^3^)
C	0.230 ± 0.005 ^a^
P2	0.200 ± 0.005 ^b^
M2	0.230 ± 0.005 ^a^
M2P2	0.240 ± 0.005 ^a^

Superscript lowercase letters indicate statistically significant differences among the samples. The acronyms C, P, M, and MP indicate the control dough and the doughs produced with pectin, marcs, and pectin and marcs, respectively.

**Table 3:** Concentration of dry matter and water in the dough samples.

Sample	Dry Matter %	Water %
C	57.9 ± 0.1 ^b^	42.1 ± 0.1 ^a^
P2	59.2 ± 0.1 ^a^	40.8 ± 0.1 ^b^
M2	58.0 ± 0.1 ^b^	42.0 ± 0.1 ^a^
M2P2	59.7 ± 0.1 ^a^	40.3 ± 0.1 ^c^

Superscript lowercase letters indicate statistically significant differences among the samples. The acronyms C, P, M, and MP indicate the control dough and the doughs produced with pectin, marcs, and pectin and marcs, respectively.

**Table 4:** Concentration of dry matter and water in the bread samples.

Sample	Dry Matter %	Water %
C	58.4 ± 0.1	41.6 ± 0.1
P2	58.4 ± 0.1	41.6 ± 0.1
M2	58.6 ± 0.1	41.4 ± 0.1
M2P2	58.4 ± 0.1	41.6 ± 0.1

ANOVA analysis evidenced no statistically significant differences among the samples. The acronyms C, P, M, and MP indicate the control bread and the breads produced with pectin, marcs, and pectin and marcs, respectively.

**Table 5:** pH and TTA of the different bread samples.

Sample	pH	TTA (meq/g)
C	5.84 ± 0.02 ^a^	0.0035 ± 0.0004 ^c^
P2	5.28 ± 0.03 ^c^	0.0065 ± 0.0002 ^b^
M2	5.34 ± 0.02 ^bc^	0.0061 ± 0.0002 ^b^
M2P2	4.88 ± 0.02 ^d^	0.0131 ± 0.0002 ^a^

Superscript lowercase letters indicate statistically significant differences among the samples. The acronyms C, P, M, and MP indicate the control bread and the breads produced with pectin, marcs, and pectin and marcs, respectively.

**Table 6:** Penetration index of the different bread samples.

Samples	Penetrometric Index (mm)
C	9.77 ± 0.71 ^a^
P2	9.49 ± 0.44 ^a^
M2	8.70 ± 0.19 ^b^
M2P2	8.81 ± 0.10 ^b^

Superscript lowercase letters indicate statistically significant differences among the samples. The acronyms C, P, M, and MP indicate the control bread, and the breads produced with pectin, marcs, and pectin and marcs, respectively.

**Table 7:** Water activity of bread crumb samples at the end of the cooling period.

Samples	a_w_ Crumb
C	0.937 ± 0.017 ^a^
P2	0.916 ± 0.001 ^bc^
M2	0.929 ± 0.005 ^a^
M2P2	0.926 ± 0.002 ^ab^

Superscript lowercase letters indicate statistically significant differences among the samples. The acronyms C, P, M, and MP indicate the control bread, and the breads produced with pectin, marcs, and pectin and marcs, respectively.

**Table 8:** Phenolic content of dough and bread samples of different recipes.

Samples	Phenolic Content (g/kg dm)	
	Dough	Bread
C	0.73 ± 0.02 ^bc^	0.67 ± 0.05 ^c^
P2	0.72 ± 0.09 ^bc^	0.78 ± 0.01 ^bc^
M2	0.92 ± 0.01 ^c^	0.92 ± 0.01 ^c^
M2P2	1.09 ± 0.01 ^a^	1.10 ± 0.01 ^a^

Superscript lowercase letters indicate statistically significant differences among the samples of dough or bread produced with the different recipes. The meanings of the acronyms are the following: C for control, P for pectin, M for marcs, and MP for pectin and marcs.

**Table 9:** Ethanol concentrations (g/kg dm) before and after leavening in different dough samples.

Sample	Ethanol Concentration (g/kg dm)		Δ% (Post-Pre)
	Pre	Post	
C	0.564 ± 0.004	0.811 ± 0.018	48.34 ^a^
P2	0.543 ± 0.008	0.798 ± 0.017	47.42 ^a^
M2	0.530 ± 0.020	0.720 ± 0.020	30.15 ^b^
M2P2	0.530 ± 0.050	0.877 ± 0.064	64.96 ^c^

Superscript lowercase letters indicate statistically significant differences among the samples. The acronyms C, P, M, and MP indicate the control dough, and the doughs produced with pectin, marcs, and pectin and marcs, respectively.

**Table 10:** Concentration of total hexose (g/kg dm) and the confidence ranges of bread samples made following the different formulae.

Samples	Total Hexose (g/kg dm)
C	2.70 ± 0.05 ^b^
P2	3.07 ± 0.01 ^a^
M2	2.43 ± 0.01 ^c^
M2P2	2.37 ± 0.21 ^c^

Superscript lowercase letters indicate statistically significant differences among the samples. The acronyms C, P, M, and MP indicate the control bread, and the breads produced with pectin, marcs, and pectin and marcs, respectively.

**Table 11:** L*, a*, b* coordinates of crumb of breads made with different recipes.

	C	P2	M2	M2P2
L*	64.265 ^b^	69.28 ^a^	41.31 ^d^	49.635 ^c^
a*	−1.065 ^b^	−1.23 ^c^	3.97 ^a^	3.73 ^a^
b*	13.465 ^a^	13.665 ^a^	7.13 ^b^	7.79 ^b^

Superscript lowercase letters indicate statistically significant differences among the samples. The acronyms C, P, M, and MP indicate the control bread and the breads produced with pectin, marcs, and pectin and marcs, respectively.

**Table 12:** Color differences (ΔE*ab) between samples at different recipes.

Samples	ΔE*ab
P2-C	2
M2-C	24
M2P2-C	16
M2-P2	26
M2P2-M2	8
M2P2-P2	18

## Data Availability

The original contributions presented in the study are included in the article, further inquiries can be directed to the corresponding authors.
